# The Loss

**Published:** 1860-03

**Authors:** 


// HALL'S JOURNAL OF HEALTH.

**Our Legitimate Scope is almost boundless: for whatever begets pleasurable and harmless feelings, promotes Health; and whatever induces disagreeable sensations, engenders Disease.**

WE AIM TO SHOW HOW DISEASE MAY BE AVOIDED, AND THAT IT IS BEST, WHEN SICKNESS COMES, TO TAKE NO MEDICINE WITHOUT CONSULTING A PHYSICIAN.

**Vol. VII.]** **MARCH, 1860.** **[No. 3.**

### THE LOSS.

WHO knows what a single hour may bring forth? of disappointment, vexation, sorrow, sickness, death, of sweetheart or pet puppy! One short hour ago, we toddled off to the printer's with the copy for the JOURNAL OF HEALTH for March in the pocket, and when nearly there, it was not in the pocket. And what does the reader suppose was the first emotion? It was, that it was providential; that most likely there were things in it which ought not to have been printed; and next, that we would return forthwith and make a better number. As for spending time in regretting any past mishap, it is sheer folly; it can do no good, and always does harm: for oftentimes the energy spent in vain regrets, or vexatious moodiness, would more than repair all the damages.

It was said of a great man that he had been engaged seven years in preparing a book for the press, and just as he had concluded the tedious task he left his study, to return in a few moments and find the manuscript burned to ashes; the light having been turned over by a favorite little dog. His only exclamation was, "O Diamond! little dost thou know the injury thou hast done!" and at once sat down to repair the loss.

Many a man would have kicked the poor little puppy out of doors, or hung him up instanter. But where would have been the philosophy of the thing? Pup "didn't go to do it!" and besides, his death would not have remedied the mischief.

A month ago we were at the *Knickerbocker* office. The discovery had just been made that the Magazine, the old-time favorite of its many thousand readers, and now the greater favorite, by reason of the life, energy, and force which the untiring industry and energy Dr. Noyes has thrown into it, had a dozen pages or more of some other publication bound in among its leaves. Here was a predicament indeed. It was the day of publication. To serve it out in that plight, would have disgraced the whole establishment. We said to the secretary, who was looking at the mischief with a pretty long face: "Didn't you get mad when you found it out?"

"Get mad! and then feel mean about it!"

Which one of our readers can lay his hand on his heart, and say that he has not, many a time and oft, got mad at some unimportant thing, and talked and blamed and scolded for "ever so long," and, when the fume and froth and fury were all gone, felt as if it would have been the most delightful retreat in the world to have crept into an auger-hole; felt so particularly mean that he could not, by any possibility, have raised courage enough to look a man in the face?

The times are numberless at which we have seen travelers, at home and abroad, on land and sea, suffering the most pitiful mortification in consequence of some out-burst of passion. Let the reader feel assured it pays well under all great emotions to say not a word. It saves conscience, saves dignity, saves self-respect. "Get mad! and then feel mean about it!" Thank you, Mr. Green, for the embodiment of an idea of such practical every-day value, and that too in brave old Saxon monosyllables:

"GET MAD! AND THEN FEEL MEAN ABOUT IT!"

